# Combined Systemic and Hepatic Artery Infusion Pump Chemo-Therapy as a Liver-Directed Therapy for Colorectal Liver Metastasis-Review of Literature and Case Discussion

**DOI:** 10.3390/cancers13061283

**Published:** 2021-03-13

**Authors:** Salman Chaudhry, Ryan C. Fields, Patrick M. Grierson, Kian-Huat Lim

**Affiliations:** 1 Comprehensive Cancer Center, Division of Oncology, Department of Internal Medicine, Barnes-Jewish Hospital and The Alvin J. Siteman, Washington University School of Medicine, St. Louis, MO 63110, USA; salman@wustl.edu (S.C.); grierson@wustl.edu (P.M.G.); 2 Comprehensive Cancer Center, Section of Surgical Oncology, Department of Surgery, Barnes-Jewish Hospital and The Alvin J. Siteman, Washington University School of Medicine, St. Louis, MO 63110, USA; rcfields@wustl.edu

**Keywords:** HAIP, FUDR, liver metastasis, colorectal cancer

## Abstract

**Simple Summary:**

Liver metastasis is a major therapeutic challenge and common cause of death for patients with colorectal cancer. While systemic treatment especially chemotherapy remains the mainstay of treatment, selected patients with liver-only metastasis may further benefit from liver-directed therapies. Direct infusion of chemotherapy into the liver metastases via an implantable hepatic arterial infusion pump (HAIP) is potentially an effective way to improve treatment response and survival in selected patients. Here, we reviewed the literature utilizing HAIP as a liver-directed modality alone and in combination with systemic chemotherapy. We discussed two cases who were successfully treated with this combinatorial approach and achieved remission or prolongation of disease control. We discussed the limitations, toxicities of combined systemic and HAIP modalities. Lastly, we provided insights on the use of HAIP in the modern era of systemic treatment for colorectal cancer patients with liver metastasis.

**Abstract:**

Colorectal cancer (CRC) is the third most prevalent malignancy and the second most common cause of death in the US. Liver is the most common site of colorectal metastases. About 13% of patients with colorectal cancer have liver metastasis on initial presentation and 50% develop them during the disease course. Although systemic chemotherapy and immunotherapy are the mainstay treatment for patients with metastatic disease, for selected patients with predominant liver metastasis, liver-directed approaches may provide prolonged disease control when combined with systemic treatments. Hepatic artery infusion pump (HAIP) chemotherapy is an approach which allows direct infusion of chemotherapeutic into the liver and is especially useful in the setting of multifocal liver metastases. When combined with systemic chemotherapy, HAIP improves the response rate, provides more durable disease control, and in some patients leads to successful resection. To ensure safety, use of HAIP requires multidisciplinary collaboration between interventional radiologists, medical oncologists, hepatobiliary surgeons and treatment nurses. Here, we review the benefits and potential risks with this approach and provide our single institution experience on two CRC patients successfully treated with HAIP in combination with systemic chemotherapy. We provide our recommendations in adopting this technique in the current era for patient with colorectal liver metastases.

## 1. Introduction

Colorectal Cancer (CRC) is the 3rd most common malignancy in the US with 149,500 new cases to be diagnosed in year 2020 [1]. It is the 2nd most common cause, accounting for 9%, of all cancer deaths [1]. Although the overall incidence and mortality of CRC has decreased over the last two decades, there has been an uptrend in detection of liver metastasis. Liver is the most common site of metastatic disease and is involved in 13–14% of the patients at initial presentation [2,3]. However, two-thirds of the patients eventually develop metachronous liver metastases during treatment or follow-up [2], which is a major cause leading to death. Therefore, limiting liver metastasis has a significant impact on progression free- and overall survival of patients with metastatic CRC.

About 70% patients with metastatic CRC have liver only metastasis at initial presentation [4]. This offers a unique opportunity for intensive liver-directed therapies to contain or stall the disease from spreading to other distant sites. Whenever possible, these patients are treated with curative intent by a multi-modality approach [5]. Resection of colorectal liver metastasis (CRLM) in combination with systemic chemotherapy has been the mainstay treatment for such patients [5]. Typically, if the metastases are initially deemed unresectable due to bulky, bilobar distribution or presence of significant extrahepatic disease, these patients would receive systemic chemotherapy followed by restaging scans for reassessment of resectability. In addition to systemic chemotherapy, the unique dual blood supply to the liver presents a unique opportunity for focused delivery of radiation [6] and chemotherapy [6] to treat liver metastasis. Specifically, the hepatic portal venous system supplies 75% of blood to the normal liver parenchyma, whereas the remaining 25% is provided by the hepatic arteries. On the contrary, blood flow to metastatic CRC tumors in the liver are almost exclusively supplied by the hepatic arteries [7]. Capitalizing on this anatomy, hepatic artery infusion pump (HAIP) chemotherapy was developed as a means to directly deliver chemotherapeutic agents into liver metastases while sparing most of the normal liver tissues. In this review, we discuss the role of HAIP in the treatment of CRLM.

## 2. Management of Resectable Liver Metastasis

Of all CRC patients who initially present with synchronous CRLM, about 20–30% have solitary or oligo-metastatic liver lesions [8]. Simultaneous resection of the primary colon cancer and synchronous liver metastases with curative intent followed by adjuvant chemotherapy offer a better overall survival [9], with one out of six patients expected to be cured during long term follow up [10]. This approach is supported by a recent prospective randomized controlled trial in which patients with resectable synchronous colorectal cancer liver metastases were randomized to simultaneous (SR) versus delayed resection (DR, 12–14 weeks) of liver metastases. At 2 years after randomization, overall survival rates and disease-free survival rates were 87.2% and 69.6% (*p* = 0.05), and 35.9% and 17.4% (*p* = 0.05) in the SR and DR groups, respectively. These trends persisted in an updated follow up of nearly four years [11]. Surgical complications associated with resection have reduced significantly over time due to improvement in surgical techniques, preoperative planning and postoperative care [12]. For example, liver parenchymal sparing approaches where major hepatectomy is replaced by multiple smaller procedure such as simultaneous liver resections, wedge resections, and ablations, has generated similar disease control rates but with significantly lower postoperative morbidity and mortality [13]. High volume specialty centers with expertise in these procedures have managed to achieve a perioperative mortality rate as low as 1% [12,14]. However, it is important to emphasize which of these approaches is the best is still debatable and treatment decision should be based on availability of expertise and experience of the treatment team and the individual patient condition.

Resection of CRLM is carefully considered for patients with a high likelihood of meeting criteria for R0 curative resection. Multiple considerations are in place and these include the ability to preserve at least two contiguous liver segments, leave enough healthy tissue (>20% in a normal liver or >30% in a liver with significant fibrosis or mild cirrhosis) to maintain adequate liver function and allow regeneration. Biliary drainage and vascular supply after negative margin resections of all lesions also needs to be considered [15,16,17,18,19]. Therefore, aggressive debulking of more extensive liver metastasis at the expense of these critical factors did not lead to better progression free or overall survival [20]. Several scoring systems have been developed to predict outcomes after resection [17,21]. For example, negative surgical margins and absence of extrahepatic disease predict higher chances of cure [9,22]. Other predictive characteristics include age, number and size of liver metastases, lymph node involvement by primary tumor and CEA level [9].

## 3. Management of Unresectable Liver Metastasis

For the remaining 70–80% of patients who have more extensive or bulky CRLMs that do not meet the criteria for upfront resection as discussed above, systemic chemotherapy is typically the first option considered. Further liver-directed therapies including portal vein embolization followed by staged liver resection, transcutaneous microwave or cryoablation, external beam radiation and arterially directed therapies which include transarterial radioembolization (TARE), transarterial chemoembolization (TACE) and hepatic artery infusion pump (HAIP) chemotherapy [23,24,25] have been developed to help control or eliminate CRLMs. However, it must be made clear that these modalities should be used in carefully selected patients, preferably under clinical trial settings and administered in conjunction with systemic chemotherapy by an experienced multidisciplinary team in order to derive the maximum benefit and minimize liver toxicities [26].

Another emerging approach to the management of unresectable CRLM is liver transplantation. Although traditionally used for hepatocellular carcinoma (HCC), a select subset of patients with isolated CRLM with favorable disease control with upfront systemic chemotherapy appear to benefit from liver transplantation as suggested by the 2013 results of the SECA study [27]. Herein 21 patients with CRLM underwent liver transplantation without adjuvant chemotherapy and demonstrated an estimated 5-year overall survival of 60%. Approximately 70% of patients recurred in the lung at a median of 6 months, yet nearly half of these had suspicious pulmonary nodules pre-transplant, suggesting that the true recurrence rate is perhaps lower. At present, liver transplantation for isolated CRLM cannot be considered standard given lack of established eligibility criteria and without prospective randomized data, yet initial observations among patients with favorable disease control on systemic chemotherapy with a good performance status suggest further investigation is warranted (reviewed in [28]).

## 4. Hepatic Artery Infusion Pump (HAIP) Chemotherapy

Hepatic artery infusion chemotherapy works by infusion of chemotherapy agents through the hepatic artery selectively into metastatic tumors while sparing the majority of the liver parenchyma from chemotherapy-related toxicities. The most commonly used chemotherapeutic agent for hepatic artery infusion is Floxuridine (FUDR). As FUDR has high first pass extraction (94 to 99 percent) and is rapidly metabolized within the tumor cells to the active metabolite, 5-FU (which has a first pass extraction rate of 19 to 55 percent) [29], FUDR produces higher intratumoral 5-FU concentration in CRLM cells with significantly lower systemic and liver toxicities [30]. Hepatic artery pump (HAIP) has been used since the early 1970s for hepatic artery infusion and has undergone significant technical evolution [31,32]. To date, implantable hepatic artery infusion pumps are preferred, as they are less likely to have catheter-related complications including dislodgment, variation in infusion rate, thrombosis and sepsis [33,34]. Modern HAIPs have also been designed to deliver continuous instead of bolus infusion, which enhances the exposure time of CRLM to FUDR and mitigates systemic toxicities [35].

Although earlier studies have shown more favorable response rates and potentially overall survival of HAIP alone versus systemic therapy [36,37,38], a later meta-analysis of ten studies showed that the increased response rate in the liver from HAIP chemotherapy did not translate into better overall survival compared to systemic chemotherapy alone [39], largely due to the improvement in the efficacy of modern systemic chemotherapy and the use of 5-FU (instead of FUDR) in several, mainly European, studies. Therefore, HAIP is now being used primarily in combination with systemic chemotherapy in most patients, as the best results occur in combination regimens [40].

In a phase I trial, the combination of HAIP FUDR with systemic irinotecan plus oxaliplatin or systemic oxaliplatin plus 5-FU/leucovorin showed impressive response rates of 90% and 87%, respectively [41]. In another phase I study, the combination of HAIP FUDR and systemic irinotecan had a slightly lower response rate of 74% [42]. In a phase II study, the combination of HAIP FUDR and systemic oxaliplatin and irinotecan demonstrated a 92% response rate (including 8% complete response), which translated into a 47% conversion to resection (57% in chemotherapy-naïve patients [43]). In more recent phase II studies, HAIP FUDR in combination with systemic FOLFOX or FOLFIRI demonstrated a response rate of 76%, and 47% of CRLM patients achieved conversion to surgical resection [44]. In this study, addition of systemic bevacizumab led to more liver toxicities with no improvement in outcome. Patients who were able to undergo surgical resection had a 3-year overall survival of 80% as opposed to 26% for those who did [44]. A meta-analysis that includes eleven studies showed a response rate was 50% for HAIP plus systemic chemotherapy and 18% rate of successful conversion to surgery. The median overall and 5-year survival for patients who underwent liver metastasectomy versus those who did not was 53 months and 49%, compared to 16 months and 3%, respectively [45]. These data show that systemic chemotherapy in combination with HAIP chemotherapy is a reasonable option in eligible patients being treated at centers where HAIP expertise is available. Addition of HAIP chemotherapy in selected patients can enhance resection rate by downstaging CRLM, which translates into prolongation of survival. It also allows for safe resection in a group of patients with initially unresectable disease.

For patients with CRLM, treatment with systemic chemotherapy followed by liver resection is able to provide long-term remission is a subset of patients. However, disease recurrence in the liver remains a common cause of ultimate failure [46,47]. Data from Memorial Sloan Kettering Cancer Center showed that addition of HAIP chemotherapy to systemic chemotherapy following hepatic resection resulted in improvement in 5-year hepatic recurrence-free survival of 62% and overall survival of 61% [48]. A larger scale retrospective analysis which included 2128 patients, of which 601 patients (28.2%) received adjuvant systemic chemotherapy with or without HAIP chemotherapy following resection of CRLM showed that patients treated with both HAIP and systemic chemotherapy had a significantly lower rate of initial intrahepatic recurrences (22.9% versus 38.4% for systemic chemotherapy only, *p* < 0.001). However, both groups of patients experienced similar cumulative 5-year overall recurrence rates (63.5% versus 64.2%, *p* = 0.74) because patients treated with HAIP and systemic chemotherapy were more likely to develop extrahepatic recurrences, predominantly in the lungs (33.6% versus 23.7% for systemic chemotherapy only, *p* < 0.001). Nonetheless, usage of HAIP chemotherapy appeared to improve overall survival (adjusted HR 0.67, 95% CI 0.57–0.78, *p* < 0.001) [49]. This study showed that patients treated with a combination of HAIP and systemic chemotherapy had a significantly improved disease-free survival (20 months with the combination as compared to 14 months with systemic therapy alone) and overall survival (median OS 84 months with the combination vs. 57 months with systemic therapy alone). Although the rate of pulmonary metastases was higher with the combined therapy, the rate of hepatic recurrences at 5 years was decreased by 15.5%, which may explain the superior DFS and OS with the combination approach. It is important to emphasize that the survival data of patients treated with HAIP chemotherapy in these studies are clearly superior to known historical data for general metastatic CRC patients treated with chemotherapy alone. This discrepancy is best explained by a likely selection bias wherein various factors including exclusion of patients with significant extrahepatic disease burden, poor performance status, patients whose disease is refractory to systemic chemotherapy, and lack of quality care provided by a multidisciplinary team in these studies. Nonetheless, addition of HAIP chemotherapy to adjuvant systemic chemotherapy can be considered for selected patients after resection of CRLM to enhance the chance for long-term remission or cure.

## 5. Two Cases Demonstrating the Benefit of HAIP Chemotherapy

### 5.1. Case 1

**Figure 1:**
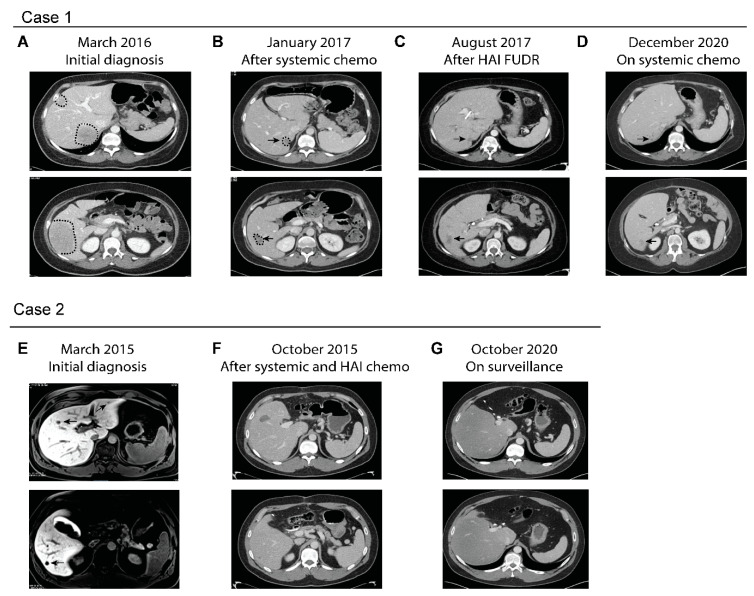
Serial imaging studies of two representative cases treated with systemic and HAIP chemotherapy. (**A**–**D**) Serial CT images of Case 1. The patient initially presented with metastatic sigmoid colon cancer with bulky disease burden in the liver (**A**). After prolonged treatment with systemic FOLFIRI and panitumumab which led to good treatment response (**B**), she subsequently underwent surgical resection of the primary colon cancer and liver lesions and placement of HAIP. (**C**) CT scans after completion of systemic and HAIP chemotherapy showed post-surgical changes (arrows) which remain unchanged over the following three years (**D**) despite extrahepatic recurrence. (**E**–**G**) Serial imaging of Case 2. (**E**) Initial MRI showing multiple lesions in Case 2 who had just undergone sigmoid colectomy. The patient was treated with 4 cycles of chemotherapy followed by left liver resection, ablation of liver lesions and placement of HAIP. (**F**) CT after completion of adjuvant systemic and HAIP chemotherapy showed post-surgical changes which remain stable over the following five years (**G**).

A 45-year-old otherwise healthy woman presented with intermittent left lower quadrant pain and decreased stool caliber over one year. A computerized tomography (CT) scan done in March 2016 showed multiple liver lesions in bilateral liver lobes, focal segmental thickening of the sigmoid colon with adjacent sigmoid mesocolon lymph nodes (Figure 1A). She subsequently underwent an ultrasound-guided liver biopsy of one of the liver lesions, which showed adenocarcinoma consistent with colorectal primary. Colonoscopy revealed a near-obstructing sigmoid colon mass, which was biopsied showing adenocarcinoma. Initial serum CEA level was 874 ng/mL. Molecular profiling of the tumor samples showed proficient MMR and no *RAS/BRAF* mutations. She was treated initially with FOLFIRI and panitumumab every two weeks, which led to rapid resolution of all her symptoms. She continued to stay on chemotherapy, which was later de-escalated to maintenance 5-FU and panitumumab. After ten months of chemotherapy, her CT scans showed marked treatment response in the liver and sigmoid colon (Figure 1B). Per tumor board recommendations she underwent sigmoid colectomy, wedge resections of liver lesions and placement of HAIP in March of 2017. Surgical pathology showed residual adenocarcinoma in the resected sigmoid colon and liver lesions. None of the twenty-three peri-colonic lymph nodes showed evidence of malignancy. She was subsequently treated with six cycles of FUDR delivered monthly via HAIP (14 days of FUDR alternating with 14 days of heparin/saline/dexamethasone), concurrently with biweekly systemic 5-FU and panitumumab. Subsequent CT scans showed no evidence of disease other than postsurgical changes in the liver. Serum CEA levels also fell to normal range. Due to malfunction, her HAIP was subsequently removed. She underwent surveillance until August 2018, when CT scans showed disease recurrence presenting as a large pelvic mass and multiple lung lesions. However, no evidence of disease was detected in the liver (Figure 1C). She subsequently underwent resection of the pelvic mass and was restarted on systemic chemotherapy, FOLFIRI plus panitumumab. Her most recent CT scans in December 2020, close to five years after initial diagnosis and two years after extrahepatic recurrence, showed stable disease in her lungs, which are the only organ affected, and no evidence of liver metastasis (Figure 1D). She continues to stay on maintenance 5-FU and panitumumab.

This is an example demonstrating the ability of HAIP chemotherapy in successfully controlling liver metastases after a prolonged course of systemic chemotherapy. As reported [49], the patient eventually did develop pelvic recurrence, which was surgically removed, and lung metastases, which are now treated with systemic chemotherapy. It is plausible to speculate that her disease course could have been more challenging and her survival shorter if she had liver metastases during the course of treatment, which may have limited her treatment options if her liver function was compromised.

### 5.2. Case 2

A 31-year-old man with no family history of colorectal or gastrointestinal cancers presented with hematochezia. A colonoscopy revealed a bleeding mass in the proximal sigmoid colon, which was biopsied showing invasive moderately differentiated adenocarcinoma. CT scans showed a sigmoid mass with regional lymphadenopathy and no evidence of distant metastasis. In February 2015, he underwent a low anterior resection with regional lymphadenectomy. Surgical pathology revealed moderately differentiated adenocarcinoma measuring 2.6 cm with 14 of resected 29 lymph nodes being involved. Molecular profiling showed the tumor had proficient MMR and *KRAS^G12D^* mutation. Unfortunately, postsurgical CT scans showed at least four suspicious new liver lesions, which were better delineated by MRI (Figure 1E). One liver lesion was later biopsied and confirmed to be metastatic moderately differentiated adenocarcinoma. Per tumor board recommendation, he underwent four cycles of FOLFOXIRI plus bevacizumab followed by exploratory laparotomy, which included left lateral segmentectomy of the liver, microwave ablation of the remaining right liver lesions and placement of an HAIP. Microwave ablation of the right liver lesions were performed, instead of hepatic resection, as a parenchyma-sparing strategy. Surgical pathology revealed metastatic adenocarcinomas in the left liver with marked treatment necrosis. After surgery, he completed 4 months of adjuvant 5-FU (oxaliplatin omitted due to allergic reaction) concurrently with 6 cycles of HAIP FUDR. Post-treatment CT scans showed no evidence of metastatic disease other than post-ablative changes in the liver (Figure 1F). He remained in remission for the following five years (Figure 1G) and is currently being followed semiannually.

This case demonstrates the potential benefit of HAIP, in conjunction with perioperative systemic chemotherapy, in a patient with multiple liver metastases. It is worth noting that his liver metastases were detected immediately after the primary colon surgery, which also yielded extensive locoregional lymph node involvement. Given the extensiveness of his disease, young age and the goal for long-tern remission, he was treated with FOLFOXIRI plus bevacizumab in order to attain the deepest possible treatment response prior to liver resection. Subsequent adjuvant HAIP and standard systemic chemotherapy was able to put him in remission for more than five years.

## 6. Limitations with the Use of HAIP

Intrathecal pain pumps from various manufacturers have been used as HAIP chemotherapy infusion pumps. For our patients, Codman HAIP with uniform infusion rate was used. Management of HAIP requires a team of trained professionals with multidisciplinary expertise ranging from careful patient selection, surgical placement of the pump, radiographic confirmation of proper arterial perfusion, safe needle access to the pump reservoir, acquisition and storage of FUDR chemotherapy, experience in anticipating and managing toxicities associated with combined systemic and HAIP chemotherapies, solving of HAIP malfunctions and management of long-term HAIP complications.

Like other specialized surgical procedures, placement of the HAIP must be performed by highly experienced surgeons [50,51]. The success rate of pump placement is higher if the surgeon has already placed more than 25 pumps [50]. A recent meta-analysis showed that institutional volume of HAIP procedure dictates the success rate of HAIP functionality after placement, which ranges from 30 to 100 percent [51]. Proper radionuclide perfusion study must be performed and interpreted by experienced interventional radiologists prior to the HAIP being cleared for clinical use [52]. Access of the HAIP reservoir, which is typically done at bedside using infusion needles, also requires proper training of the nursing staff to obtain accurate record of remnants prior to each instillation, as well as to minimize complications such as bleeding, infection, patient discomfort and FUDR extravasation. Lastly, medical oncologists with knowledge and experience in managing both systemic and HAIP chemotherapy are critical in adjusting the dosages of each chemotherapeutics in order to avoid excessive or irreversible liver toxicities.

Patient selection is critical in decision making for HAIP placement and should be conducted in multidisciplinary tumor board conferences. Significant extrahepatic disease is a relative contraindication to placement of HAIP. As anti-VEGF antibodies such as bevacizumab should not be administered concurrently with HAIP chemotherapy due to excessive liver toxicities, control of extrahepatic disease is expected to be compromised, which defeats the purpose of HAIP chemotherapy. To be deemed safe for HAIP chemotherapy, which is expected to cause collateral damage to the normal liver parenchyma, patients should have adequate hepatic reserve (relative contraindication of bilirubin > 1.5 mg/dl or ascites) and the tumor should involve less than 70% of liver, which ideally should be determined by computerized liver volumetry using 3-dimensinal MRI. For patients who have known underlying liver disease or radiographic evidence of cirrhosis or steatosis, a biopsy of the liver parenchyma is helpful in surgical planning. Presence of portal vein thrombosis is a relative contraindication [35]. Equally importantly, because HAIP requires regular, timely re-instillation of FUDR or normal saline, patients will need to be evaluated by the treatment team on a regular basis, typically every two to three weeks without fail in order to prevent HAIP malfunction. Therefore, usage of HAIP should only be considered for patients who are highly committed and clearly understand the risks and benefits of this approach.

The most significant long-term complication associated with HAIP is biliary sclerosis, which has been reported in 2% of patients with unresectable metastatic disease treated with HAIP FUDR only and in 5.5% of patients receiving adjuvant HAIP therapy after hepatectomy [53]. Presence of biliary sclerosis, most frequently in the hepatic ducts, is significantly associated with more frequent infections (50.0% vs. 14.8% of patients without HAIP). However, biliary sclerosis is itself not associated with higher mortality [53] if adequately salvaged with stenting and dilation of the affected ducts. As the levels of biliary sclerosis can be multifocal and distant from the ampulla of Vater, experienced interventional gastroenterologists and/or radiologists are frequently needed to successfully perform these procedures.

## 7. Potential Role of HAIP in the Modern Era of Systemic Treatment

Systemic therapies for CRC have greatly evolved in recent years, largely attributed to the advancement of genomics and personalized medicine. Perhaps the greatest value of HAIP, in the older era when chemotherapeutics was limited to 5-FU, oxaliplatin and irinotecan, was to improve response rate in CRLM to enable hepatectomy or metastasectomy. For patients who were unable to undergo successful liver resections, HAIP chemotherapy may prolong progression-free survival or overall survival in selected cases. However, recent evaluation of the systemic treatment options for CRC now warrants further investigation into the role of HAIP chemotherapy.

First, escalation of the strength of systemic chemotherapy has been shown to significantly improve response rate and progression free survival. The combination of oxaliplatin, irinotecan, 5-FU and leucovorin (FOLFOXIRI) plus bevacizumab demonstrated a 65% response rate compared to 53% with FOLFIRI plus bevacizumab [54]. The effect of FOLFOXIRI and bevacizumab was similar in all subgroups regardless of baseline clinical characteristics or *RAS/BRAF* mutational status [55]. In a multinational randomized phase II trial (OLIVIA trial) conducted in Europe, FOLFOXIRI plus bevacizumab administered to patients with initially unresectable CRLM demonstrated an objective response rate of 81%, with 61% of treated patients being converted to resection [56]. R0 resection was achieved in 49% of patients treated with FOLFOXIRI plus bevacizumab. In both studies, FOLFOXIRI plus bevacizumab was associated with higher toxicities including diarrhea and bone marrow suppression. In a phase II (VOLFI) study, the combination of FOLFOXIRI plus panitumumab achieved an objective response rate of 87.3% as opposed to 60.6% with FOLFOXIRI alone for patients with treatment naïve RAS wild-type metastatic CRC. Secondary resection was achieved in 33.3% of patients treated with FOLFOXIRI plus panitumumab, as opposed to 12.1% in the FOLFOXIRI group [57]. Given the high response rates of FOLFOXIRI-based regimens, it is unclear whether adding HAIP chemotherapy as an adjunct would further improve the conversion rate in selected patients. However, liver toxicities are a major concern especially since significant liver resections are being planned in later stage and therefore the proper dosage of each agent should be carefully evaluated in clinical trials. Again, it should be cautioned that bevacizumab should not be co-administered concurrently with HAIP chemotherapy due to excessive liver toxicities. However, combining HAIP FUDR with systemic FOLFOXIRI plus panitumumab could be a more effective approach and should be investigated in clinical trials.

Another major class of therapy that is now available for patients with metastatic CRC is immune checkpoint inhibitors (ICIs), which include anti-PD1 (pembrolizumab and nivolumab) and anti-CTLA4 (ipilimumab) antibodies. Pembrolizumab is now FDA-approved for patients with high microsatellite instability (MSI-H) /mismatch repair deficient (dMMR) or high tumor mutational burden (TMB) CRC tumors. About 15% of all CRC tumors are MSI-H or dMMR. These tumors contain hereditary or somatic loss-of-function mutations or epigenetic silencing of one or more of the mismatch repair genes that are critical in DNA repair [58]. As a result, the tumor cells harbor high mutational burdens and are more likely to produce neoantigens that are recognized by the host immune system as foreign entities. Therefore, administration of immune checkpoint inhibitors unleashes host immunity, leading to tumor rejection. In the frontline setting, pembrolizumab achieved a 43.8% response rate, as opposed to 33.1% in the chemotherapy group [59]. Progression free survival among patients treated with pembrolizumab was 16.5 months as opposed to 8.2 months in the chemotherapy group. Importantly, grade 3 or higher treatment-related adverse events occurred in 22% of the patients in the pembrolizumab group, as compared with 66% (including one patient who died) in the chemotherapy group. Pembrolizumab demonstrates a 40% response rate even in patients with chemotherapy-refractory MSI-H/dMMR CRC [60]. Furthermore, ipilimumab plus nivolumab demonstrated a 51% objective response rate for patients with MSI-H/dMMR CRC pretreated with at least one line of chemotherapy [61]. These data clearly show ICI to be superior to standard chemotherapy from both the efficacy and safety perspectives in MSI-H/dMMR CRC patients regardless of treatment status. Inclusion of HAIP chemotherapy with ICI has not been studied but is certainly intriguing. As a locoregional therapy, HAIP is not expected to be overly immunosuppressive and blunt the effect of ICI. On the contrary, the tumoricidal effect of HAIP FUDR may release more tumor neoantigens into the circulation or lymphatic system, which could further prime host anti-tumor immunity and potentiate the effect of ICI. Therefore, it would be interesting to determine whether addition of HAIP to ICI could deepen and/or increase the response rate of ICI alone beyond the current 40–50% range and successfully convert more patients with CRLM to resection.

## 8. Conclusions

HAIP was developed as an effective treatment tool in the era when systemic chemotherapy was not as effective. As systemic treatment has become increasingly personalized and more effective, selection of patients who would benefit from HAIP has become increasingly critical. Incorporation of HAIP into modern systemic chemotherapy or immunotherapy regimens should be further investigated in clinical trials. It must be emphasized that unlike most other liver-directed approaches, once placed, HAIP requires continued care by a multidisciplinary team of experts and patients. Due to the high level of care demanded, HAIP at its current stage is not expected to be widely adopted in the community but instead should be offered in high volume centers. However, when carefully planned, HAIP plus systemic treatment remains a viable and effective option that can realistically offer a chance for cure in selected patients.
